# Population-Based Incidence of Guillain-Barré Syndrome During Mass Immunization With Viral Vaccines: A Pooled Analysis

**DOI:** 10.3389/fimmu.2022.782198

**Published:** 2022-02-03

**Authors:** Fengge Wang, Donglan Wang, Yingjie Wang, Cancan Li, Yulu Zheng, Zheng Guo, Pengcheng Liu, Yichun Zhang, Wei Wang, Youxin Wang, Haifeng Hou

**Affiliations:** ^1^ School of Public Health, Shandong First Medical University and Shandong Academy of Medical Sciences, Taian, China; ^2^ Centre for Precision Health, School of Medical and Health Sciences, Edith Cowan University, Perth, WA, Australia; ^3^ Beijing Key Laboratory of Clinical Epidemiology, School of Public Health, Capital Medical University, Beijing, China

**Keywords:** Guillain-Barré syndrome, virus, vaccine, mass immunization, systematic review, meta-analysis

## Abstract

Misunderstanding temporal coincidence of adverse events during mass vaccination and invalid assessment of possible safety concerns have negative effects on immunization programs, leading to low immunization coverage. We conducted this systematic review and meta-analysis to identify the incidence rates of GBS that are temporally associated with viral vaccine administration but might not be attributable to the vaccines. By literature search in Embase and PubMed, we included 48 publications and 2,110,441,600 participants. The pooled incidence rate of GBS was 3.09 per million persons (95% confidence interval [CI]: 2.67 to 3.51) within six weeks of vaccination, equally 2.47 per 100,000 person-year (95%CI: 2.14 to 2.81). Subgroup analyses illustrated that the pooled rates were 2.77 per million persons (95%CI: 2.47 to 3.07) for individuals who received the influenza vaccine and 2.44 per million persons (95%CI: 0.97 to 3.91) for human papillomavirus (HPV) vaccines, respectively. Our findings evidence the GBS-associated safety of virus vaccines. We present a reference for the evaluation of post-vaccination GBS rates in mass immunization campaigns, including the SARS-CoV-2 vaccine.

## Introduction

The coronavirus disease-2019 (COVID-19), induced by the severe acute respiratory syndrome coronavirus-2 (SARS-CoV-2), has been challenging all over the world since December, 2019 (1). As of December 29, 2021, the total number of confirmed cases is over 281 million worldwide, including more than five million deaths (2). SARS-CoV-2 infection is commonly characterized by fever, cough, shortness of breath, headache fatigue, pneumonia and congestion (3, 4). In severe cases, especially among individuals over 60 years old and those with underlying chronic comorbidities, the infection leads to acute respiratory distress syndrome (ARDS), renal failure, meningoencephalitis, cerebrovascular accidents, sepsis and even death (5). Compared with its predecessors (*i.e.*, SARS-CoV and MERS-CoV), SARS-CoV-2 transmits much more efficiently from person to person (6).

Currently, mass vaccine immunization is believed essential to control the pandemic (7). The SARS-CoV-2 vaccine has been expedited through preclinical and clinical investigations (8). As of December 29, 2021, a total of 8,687,201,202 vaccine doses have been administered worldwide (2). Efforts have been made to promote mass vaccination programs against SARS-CoV-2. The unprecedented campaign of mass immunization will pose many challenges to the assessment of vaccine safety. Potential adverse events following immunization (AEFI), induced by vaccination of SARS-CoV-2, will foreseeably raise potential concerns under the pandemic. The public needs frequent reassurance of vaccine safety when adverse events occur in temporally coincident association with SARS-CoV-2 vaccination, even when the events are not caused by the vaccines. Awareness of possible adverse events is essential for the assessment of vaccine safety and may help to separate AEFI from events that are temporally associated with but might not be attributed to vaccination (9).

Viral vaccines were considered to be related to AEFIs including vomiting, diarrhea, nausea or abdominal pain, acute otitis media, vaccine-related paralytic poliomyelitis (VAPP), Guillain-Barré syndrome (GBS), anaphylactic shock, epilepsy and meningitis (10), among which GBS is considered one of the most severe conditions (11). GBS is featured by immune mediators damaging to peripheral nerves and associated with muscle weakness or paralysis (12). The initial symptoms of GBS are severe nerve pain in the neck, shoulder and waist, followed by acute progressive acute paralysis of limbs and subjective sensory disturbance (13). Reported incidence rates of GBS for all ages combined range from 0.2 to 3.0 per 100,000 person-years (14).

An 11- to 18-fold increase of incidence rate of GBS within three weeks after influenza vaccination and a 4- to 9-fold increase within six weeks have been released previously (12). Consideration about GBS that was in the wake of post-vaccination appeared for the first time in the influenza vaccine season from 1976 to 1977 (15). Mass human papillomavirus (HPV) immunization has also been suggested to be related to GBS (16). At present, HPV vaccines are recommended by World Health Organization (WHO) for girls between 9 and 13 years old (17, 18).

Recently, an 82-year-old female developed GBS two weeks after receipt of the first dose of Pfizer COVID-19 vaccine (19). Thereby, rational interpretation of GBS occurrence temporally associated with vaccination is needed to the public. A valid interpretation of the coincidental adverse events may prevent from misunderstanding such reports, and contribute to the acceptance of vaccination campaigns.

In order to identify the incidence rate of GBS in populations that received viral vaccines during mass immunization campaigns, we conducted this systematic review and meta-analysis. We expected to provide the reference for the public to assess post-vaccination GBS validly, and engage in averting potential spurious association between the vaccine and coincidental adverse events during the mass vaccination against SARS-CoV-2.

## Materials and Methods

This study was conducted by reference to the Preferred Reporting Items for Systematic Reviews and Meta-Analyses (PRISMA) guideline (20), which is provided in **Table S1**.

### Literature Search Strategy

We performed a systematic literature search on Embase and PubMed databases to identify all relevant studies published up to December 31, 2020. The search strategy was based on the combination of the following terms: “Guillain-Barré syndrome”, “Guillain-Barre syndrome”, “acute infectious polyneuritis”, “acute inflammatory demyelinating polyneuropathy”, “Landry-Kussmaul syndrome”, “Landry-Guillain-Barré syndrome”, “Landry′s syndrome”, “Kussmaul-Landry syndrome”, “Landry′s paralysis”, “vaccine”, “vaccination”, “inoculation”, “immunize”, “vaccinum”, “bacterin”, “immunization”, “immunise”, “immune”, “vaccines”. References cited in the included articles were also screened to find additional studies.

### Literature Screening and Selection

Firstly, the titles and abstracts of the publications were reviewed by two authors (FW and DW) independently. Secondly, the full text and online supplementary data were read to determine the eligibility of the publications. Any uncertainties and discrepancies were resolved by discussion with the third author (YW). The inclusion criteria were: 1) studies that reported temporal coincidence of GBS in mass immunizations; 2) participants received viral vaccines, including but not limited to influenza vaccine, HPV vaccine, polio vaccine, hepatitis vaccine, measles-rubella vaccine, rubella vaccine, or measles-mumps-rubella (MMR); 3) the following data were available or can be calculated: number of GBS patients, number of vaccinated populations, or background rate of GBS after vaccination. The quality of the literature was assessed by two authors (CL and YW) independently (**Table S2**).

Studies matching the following items were excluded: 1) reviews, case report studies, letters and conference abstracts; 2) animal studies; 3) clinical studies evaluating the safety of vaccines; 4) studies did not provide the information of vaccines in detail; 5) studies with the duplicate publication or overlapping data.

### Data Extraction

Two authors collected the following data independently: 1) first author’s name; 2) publication year; 3) characteristic of patients (*e.g.*, ethnicity, region, gender, age-range); 4) information of vaccines (*e.g.*, the target viruses of vaccines, type of vaccine, follow-up duration after vaccination, the valence of vaccines and adjuvants of vaccines); 5) the number of study participants; 6) background rate and/or number of coincident cases of GBS during vaccination; 7) sources of vaccination. If there were duplicate data, the studies with larger sample size or newly published ones were involved.

### Statistical Analysis

The meta-analysis, a statistical procedure for the combination of the results from multiple independent studies, was performed using STATA version 16.0 (STATA Corp, College Station, TX, USA) and/or R version 4.0.4 (Foundation for Statistical Computing, Vienna, Austria), by which the pooled background rate and its 95% confidence interval (CI) were calculated. Cochran’s Q-test and *I* ^2^ statistics were applied to measure the significance of heterogeneity across eligible studies. Heterogeneity was assumed insignificant if *P* > 0.05 and *I* ^2^ < 50%, then a fixed-effect model meta-analysis was carried out, otherwise, heterogeneity was considered statistically significant, then the Der-Simonian Laird’s random-effect model was implemented (21). Moreover, subgroup analyses were conducted on the basis of gender, age range, ethnicity, target virus, type of vaccines, follow-up duration after vaccination, the valence of vaccines and adjuvants of vaccines. To estimate the stability of the pooled results and distinguish the potential influence of individual studies, a sensitivity analysis was conducted by sequential removal of every single study one at a time. In addition, the publication bias was modeled by the funnel plots and analyzed by the Egger’s test. Furthermore, the trim-fill method was used to adjust for publication bias when it is significant. A *P* < 0.05 was considered significant if not mentioned specifically.

## Results

### Literature Search

A total of 2,201 publications (1,081 from PubMed and 1,120 from Embase) were retrieved, among which 943 duplicate records were excluded. After reviewed by titles and abstracts of the remained studies 1,124 publications were excluded for the following reasons: 188 studies were reviewed, case report studies, letters and conference abstracts; 102 were no-human-based researches; 179 were clinical trials; 226 were not studies on the incidence of GBS in vaccinees; 305 were not studies on virus vaccines; and 124 were researches on the mechanism of GBS. Among the 134 articles evaluated by full-text, 64 were excluded due to no details of vaccines or doses; 17 were excluded due to no number of coincident cases of GBS during vaccination; 5 were excluded due to duplicate data.

**Figure 1:**
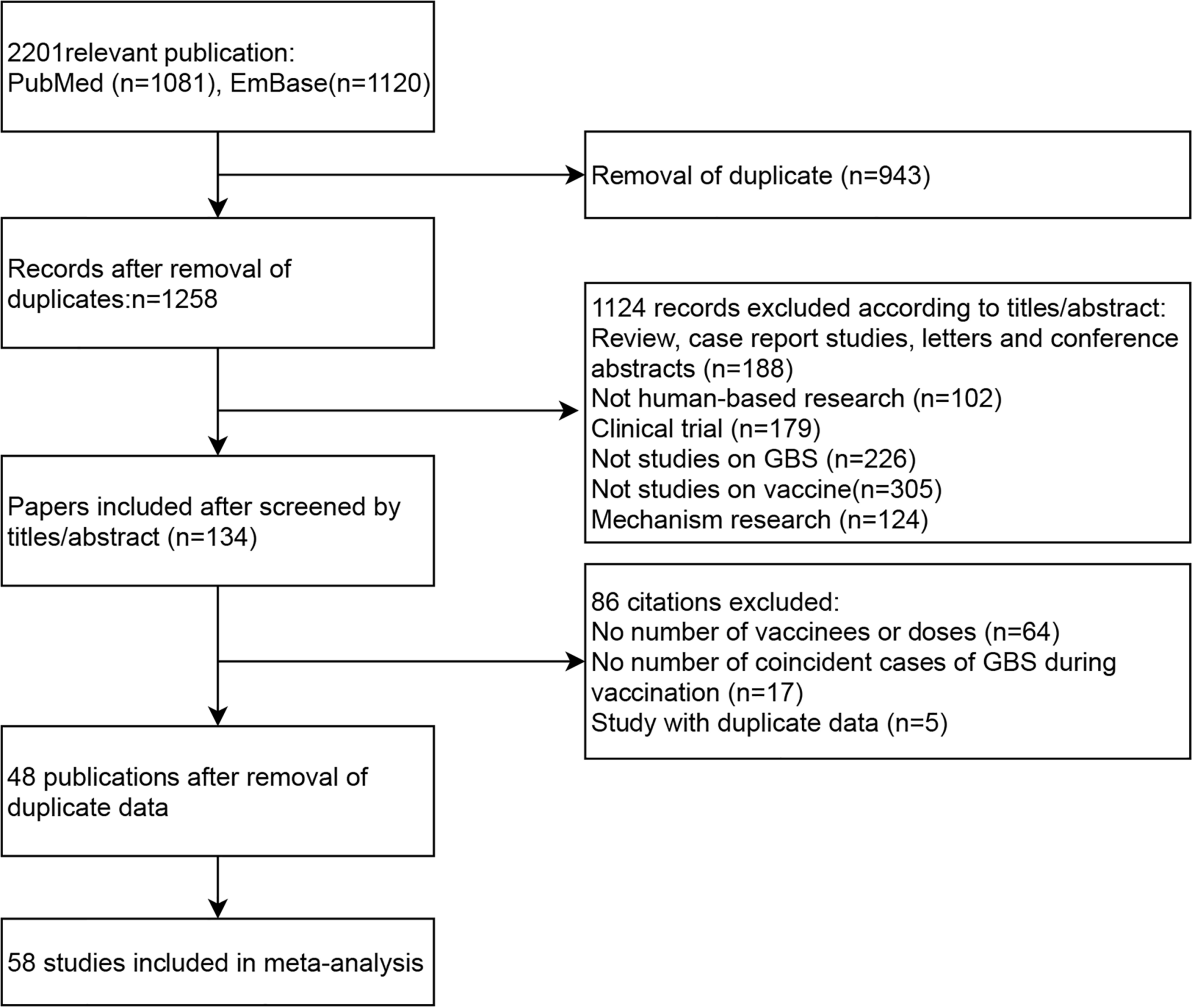
Flow diagram of study selection.

We checked the database of original studies on GBS after vaccination, and excluded one study of seasonal influenza vaccine in the U.S (22), one H1N1 vaccine study in the U.S (23), one HPV vaccine study in the U.S (24), and two studies of H1N1 vaccine in China due to duplicate data (25, 26). Finally, 48 publications with 58 independent studies were included in our meta-analysis (12, 27–74). The flow chart of literature screening process is shown in **Figure 1**.

### Characteristics of Included Studies

Of the 58 studies, 45 reported the incidence of GBS for influenza vaccines, seven reported HPV vaccines, one reported polio vaccine, one reported hepatitis vaccine, two reported Measles-Rubella vaccines, one reported Rubella vaccine, one reported measles-mumps-rubella vaccine (MMR) vaccine. There are 20 studies on inactivated virus vaccines, seven on live-attenuated vaccines, one on the recombinant vaccine and one on the split-virion vaccine. With regard to the duration of follow-up, 32 studies reported the background rate of GBS within six weeks after vaccination. The details are listed in **Table 1**.

**Table 1:** Characteristics of included studies.

Author	Publication year	Country	Target virus	Vaccine	Type of vaccine	Time of immunization	Follow-up Duration	N. of participants	N. of GBS
Lee (8)	2020	Korea	IV	TIV	NA	2014-2016	0-90 d	10,100,000	74
Phillips (61)	2020	Australia	HPV	HPV vaccine	NA	2007-2017	NA	9,400,000	5
Mauro (52)	2019	Brazil	HPV	HPV vaccine	Recombinant vaccine	2014-2016	NA	3,390,376	2
Deceuninck (36)	2018	Canada	HPV	HPV vaccine	NA	1999-2014	NA	559,988	4
Miranda (56)	2017	France	HPV	HPV vaccine	NA	2008-2012	≥1 d	842,120	20
Gee (39)	2017	U.S.	HPV	HPV vaccine	NA	2006-2015	15 d	1,708,075	1
Sandhu (65)	2017(a)	U.S.	IV	IV vaccine	NA	2010-2011	0-42 d	14,052,724	88
Sandhu	2017(b)	U.S.	IV	IV vaccine	NA	2011-2012	0-42 d	15,474,830	75
Sandhu	2017(c)	U.S.	IV	IV vaccine	NA	2012-2013	0-42 d	16,220,362	87
Sandhu	2017(d)	U.S.	IV	IV vaccine	NA	2013-2014	0-42 d	16,189,929	89
Ghaderi (40)	2016	Norway	IV	IV vaccine	Inactivated vaccine	2009	0-42 d	1,896,455	8
Tasher (69)	2016	Israel	Polio	bOPV	NA	2013-2014	23 d, 45 d, 38 d	943,587	3
Mayet (53)	2015	France	IV	TIV	Inactivated vaccine	2011-2012	4 d	256,666	1
Haber (45)	2014	U.S.	IV	TIV	Live attenuated vaccine	2005-2013	0-73 d	14,221,122	14
Kawai (49)	2014(a)	U.S.	IV	TIV	Inactivated vaccine	2012-2013	0-42 d	2,832,064	14
Kawai	2014(b)	U.S.	IV	IV vaccine	Live attenuated vaccine	2012-2013	0-42 d	187,497	1
Baxter (30)	2013	U.S.	IV	TIV	Inactivated vaccine	1994-2006	0-42 d	5,251,544	18
McCarthy (55)	2013(a)	Canada	IV	IV vaccine	Inactivated and live attenuated	2009-2010	0-84 d	538,257	9
McCarthy	2013(b)	Canada	IV	TIV	Inactivated vaccine	2009-2010	0-84 d	998,881	18
McCarthy	2013(c)	Canada	IV	TIV	Inactivated vaccine	2010-2011	0-84 d	1,158,932	28
Polakowski (62)	2013	U.S.	IV	MIV	Inactivated vaccine	2009-2010	≥1 d	3,436,452	34
Greene (42)	2013	U.S.	IV	IV vaccine	Inactivated vaccine	2009-2011	0-141 d	4,066,533	72
Choe (34)	2011	Korea	IV	TIV	Inactivated vaccine	2003-2010	1-105 d	75,000,000	9
De Wals (35)	2012	Canada	IV	MIV	NA	2009-2010	0-56 d	4,067,340	25
Souayah (67)	2012	U.S.	HBV	HBV vaccine	NA	1990-2009	≥1 d	55,588,000	189
Souayah (67)	2012(a)	U.S.	IV	MIV	Inactivated vaccine	2009	≥1 d	99,366,920	62
Souayah (68)	2012(b)	U.S.	IV	IV vaccine	NA	2009	≥1 d	53,708,996	57
Yih (74)	2012	U.S.	IV	MIV	Inactivated vaccine	2009-2010	0-70 d	2,880,797	5
Wise (73)	2012	U.S.	IV	IV vaccine	NA	2009-2010	≥1 d	32,000,000	411
Choe (34)	2011	Korea	IV	MIV	NA	2009-2010	≥1 d	17,570,000	22
Liang (51)	2011	China	IV	IV vaccine	Split-virion vaccine	2009-2010	<80 d	89,600,000	8
Mayet (54)	2011	French	IV	MIV	Inactivated vaccine	2009-2010	22 d	49,138	1
Souayah (66)	2011(a)	U.S.	IV	IV vaccine	NA	2006-2009	≥1 d	173,000,000	166
Souayah	2011(b)	U.S.	HPV	HPV vaccine	NA	2006-2009	≥1 d	8,600,000	69
Vidal (72)	2011	Mexico	IV	IV vaccine	NA	2009-2010	0-42 d	45,490,501	14
Banzhoff (29)	2011	European	IV	MIV	Inactivated vaccine	2009-2010	0-42 d	11,000,000	22
Vellozzi (70)	2010	U.S.	IV	MIV	NA	2009-2010	0-42 d	82,400,000	99
Burwen (12)	2010	U.S.	IV	TIV	Inactivated vaccine	2000-2001	0-98 d	22,200,000	238
Vellozzi (71)	2009	U.S.	IV	TIV	Inactivated vaccine	1990-2005	NA	747,070,979	581
Nakayama (59)	2007(a)	Japan	IV	IV vaccine	Inactivated vaccine	1994-2004	NA	38,020,000	9
Nakayama	2007(b)	Japan	Rubella virus	Rubella vaccine	Live attenuated vaccine	1994-2004	NA	4,000,000	1
Bino (32)	2003	European	Measles and rubella viruses	Measles-rubella vaccine	Live attenuated vaccine	1991-2001	NA	867,000	1
Patja (60)	2001	Finland	Measles, mumps and rubella viruses	MMR	NA	1982-1986	≥1 d	630,000	20
Hurwitz (47)	1981	U.S.	IV	IV vaccine	NA	1978-1979	0-56 d	12,500,000	13
Safranek (64)	1991	U.S.	IV	IV vaccine	NA	1976	0-42 d	3,822,370	45
Greene (41)	2012(a)	U.S.	IV	IV vaccine	NA	2009-2010	0-127 d	1,480,135	31
Greene	2012(b)	U.S.	IV	TIV	NA	2009-2010	0-84 d	1,724,570	39
Moro (58)	2020	U.S.	IV	TIV	Inactivated vaccine	2011-2019	≥1 d	113,100,000	61
Huang (46)	2012	China	IV	IV vaccine	Inactivated vaccine	2009-2010	≥1 d	5688517	19
Álvarez (63)	2015	Latin U.S. and Caribbean	IV	IV vaccine	NA	2009-2010	0-45 d	143,835,616	105
Arya (28)	2019	U.S.	IV	IV vaccine	NA	2015-2016	0-42 d	13,366,005	95
Andrews (27)	2017	U.K.	HPV	HPV vaccine	NA	2007-2016	0-91 d	10,400,000	9
Haber (44)	2016	U.S.	IV	QIV	Inactivated vaccine	2013-2015	0-24 d	70,000,000	13
Haber (43)	2015	U.S.	IV	QIV	Live attenuated vaccine	2013-2014	7 d, 9 d	12,700,000	2
Benedetti (31)	2015	Italy	IV	IV vaccine	NA	2010-2011	≥1 d	19,846,068	365
Moro (57)	2015	U.S.	IV	TIV	Inactivated vaccine	2013-2015	6 d, 9 d, 22 d	5,600,000	4
Esteghamati (37)	2008	Iran	Measles, mumps, rubella	MMR	NA	2002-2004	≥1 d	7,042,254	25
Izurieta (48)	2005	U.S.	IV	TIV	Live attenuated vaccine	2003-2005	≥1 d	2,500,000	2

HPV, human papilloma virus; MIV, monovalent influenza vaccine; QIV, quadrivalent influenza vaccine; TIV, trivalent influenza vaccine; IV, influenza virus; MMR, measles-mumps-rubella vaccine; N, number; NA, not available.

### Pooled Results of Post-Vaccination GBS Rate

**Figure 2:**
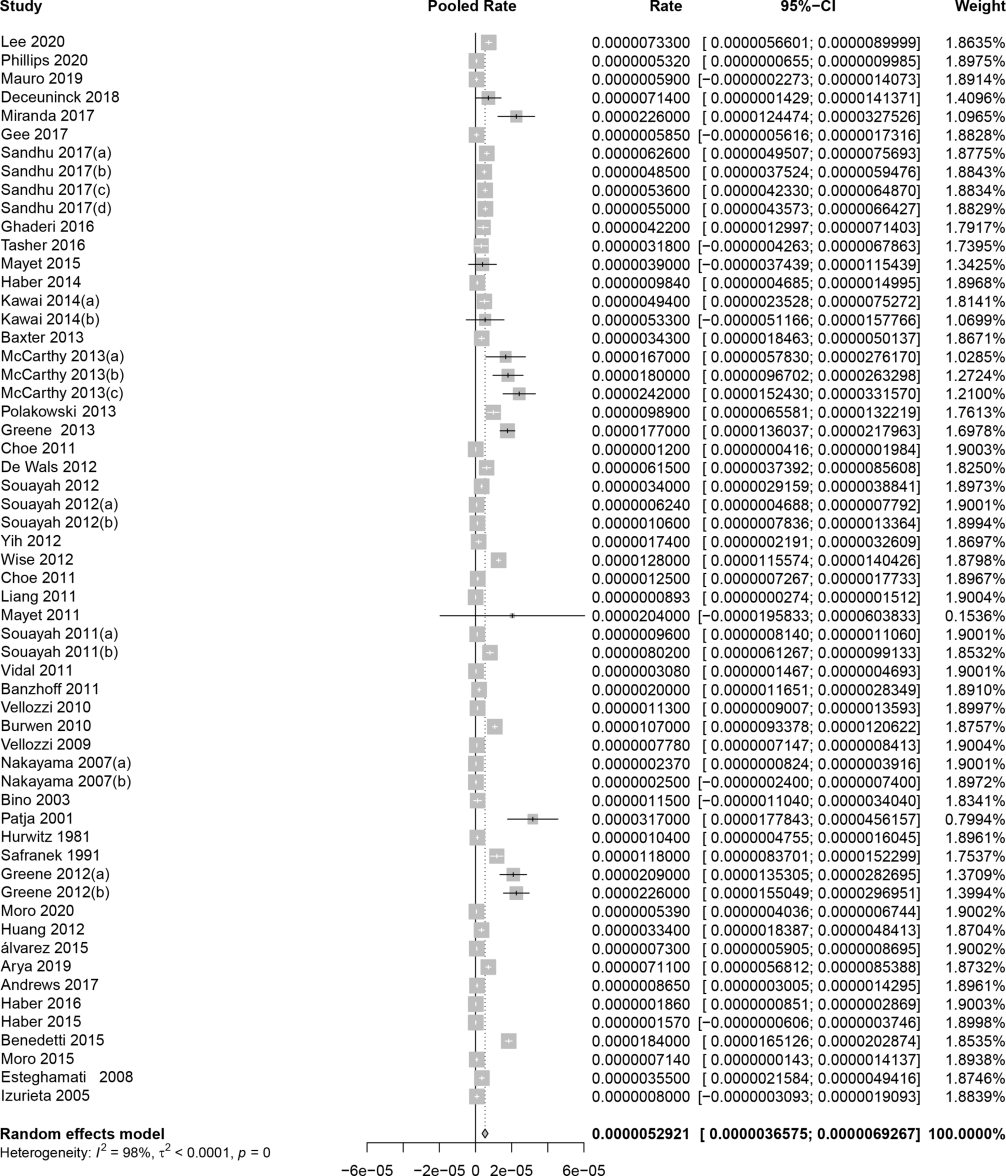
Pooled background rates of Guillain-Barré syndrome during mass immunization. CI, confidence interval.

As shown in **Figure 2**, the pooled GBS rate, synthesized by a random-effects model, was 5.29 per million (95% CI: 3.66 to 6.93 per million) after immunization of viral vaccines. The heterogeneity test showed significant heterogeneity between studies (*I^2^* = 98%, *P* < 0.01). Since the time period between 0 and 6 weeks is considered as the risk window after vaccination, we evaluated the GBS rate in this period. As result, the pooled rate was 3.09 per million persons (95% CI: 2.67 to 3.51 per million) for the 42-day window, equally 2.47 per 100,000 person-year (95%CI: 2.14 to 2.81 per 100,000 person-year). In contrast, as shown in **Table S3**, the previous studies that estimated incidence rates of GBS within general populations showed a range from 0.42 to 2.42 per 100,000 per person-year, meaning that there was no significant increase in GBS among population received viral vaccines.

### Subgroup Analyses

Subgroup analyses were performed on the basis of gender, age, ethnicity, target virus, type of vaccines, the valence of vaccines and adjuvants of vaccines. As shown in **Table 2**, the pooled incidence rates of GBS were 7.26 per million (95%CI: 3.11 to 11.41 per million) among people aged <18 years, 0.99 per million (95%CI: 0.24 to 1.73 per million) among people aged 18 to 59 years, and 6.06 per million (95%CI: 2.51 to 9.61 per million) among people aged ≥ 60 years of age. The pooled rates were 6.31 per million (95%CI: 0.81 to 11.82 per million) among men, and 6.41 per million (95%CI: 2.53 to 10.30 per million) among women. The pooled GBS rates were 5.89 per million (95%CI: 4.05 to 7.72 per million) among Caucasian vaccinees, and 0.61 per million (95%CI: 0.32 to 0.91 per million) among Asian vaccinees.

**Table 2:** Subgroup analysis and trim-fill analysis of GBS incidence (per million persons).

Subgroup		N of studies	N of participants	Pooled rate and 95% CI	*I^2 (^*%)	Egger’s test (*P*)	Adjusted pooled rate and 95% CI*
Total		58	2,110,441,600	5.29 (3.66, 6.93)	98	6.90 (<0.001)	1.71 (0, 3.96)
Duration of follow-up	Six weeks after vaccination	32	817,821,271	3.09 (2.67, 3.51)	96	8.11 (<0.001)	1.89 (1.48, 2.30)
Target virus							
	HPV	7	34,900,559	2.44 (0.97, 3.91)	92	2.55 (0.051)	2.10 (0.54, 3.66)
	IV	45	2,006,470,200	2.77 (2.47, 3.07)	98	6.45 (<0.001)	1.39 (1.07, 1.72)
	MR	2	7,909,254	2.52 (0.19, 4.85)	68	NA	NA
Valence of vaccines							
	Monovalent	9	252,770,647	3.98 (2.65, 5.32)	98	2.24 (0.060)	3.95 (2.63, 5.28)
	Trivalent	14	1,000,233,168	1.94 (1.46, 2.41)	97	2.62 (0.022)	0.98 (0.47, 1.49)
	Quadrivalent	2	82,700,000	0.18 (0.09, 0.27)	0	NA	NA
Type of vaccines							
	Inactivated vaccine	20	1,209,873,878	5.01 (2.29, 7.73)	97	2.96 (0.008)	1.05 (0, 4.72)
	Live-attenuated vaccine	7	116,875,619	0.68 (0.17, 1.20)	86	0.36 (0.730)	0.65 (0.15, 1.14)
	Split-virion vaccine	1	89,600,000	0.09 (0.03, 0.15)	NA	NA	NA
	Recombinant vaccine	1	3,390,376	0.59 (0, 1.41)	NA	NA	NA
Adjuvants							
	AS03	3	6,012,933	5.40 (3.54, 7.26)	0	0.54 (0.687)	5.37 (3.51, 7.22)
	MF59	1	11,000,000	2.00 (1.17, 2.83)	NA	NA	NA
Ethnicity							
	Caucasian	51	1,870,463,083	5.89 (4.05, 7.72)	97	6.19 (<0.001)	1.90 (0, 4.41)
	Asian	7	239,978,517	0.61 (0.32, 0.91)	95	3.80 (0.013)	0.46 (0.11, 0.82)
Age							
	<18	6	10,884,949	7.26 (3.11, 11.41)	86	2.37 (0.077)	NA
	18~59	3	279,909,637	0.99 (0.24, 1.73)	80	4.77 (0.131)	NA
	≥60	5	376,783,923	6.06 (2.51, 9.61)	99	3.75 (0.033)	4.33 (1.42, 7.25)
Gender							
	Men	3	10,541,605	6.31 (0.81, 11.82)	93	3.08 (0.200)	NA
	Women	5	25,618,567	6.41 (2.53, 10.30)	94	3.27 (0.047)	NA

*Trim-fill analysis; CI, confidence interval; HPV, Human papilloma virus; IV, Influenza vaccine; bOPV, Bivalent oral polio vaccine; MR, Measles-rubella; MMR, Measles-mumps-rubella; N, number; NA, not applied.

Based on 29 original studies reported vaccines types, the pooled GBS rates were 5.01 per million (95%CI: 2.29 to 7.73 per million) for inactivated viral vaccine, 0.68 per million (95%CI: 0.17 to 1.20 per million) for the live-attenuated vaccine, respectively. The pooled background rates of GBS were 2.77 per million (95%CI: 2.47 to 3.07 per million) for individuals received influenza vaccine, and 2.44 per million (95%CI: 0.97 to 3.91 per million) for those received HPV vaccine. In addition, the pooled background rates were 3.98 per million (95% CI: 2.65 to 5.32 per million) for monovalent vaccines of influenza vaccine, 1.94 per million (95% CI: 1.46 to 2.41 per million) for trivalent vaccines of influenza, and 0.18 per million (95% CI: 0.09 to 0.27 per million) for quadrivalent vaccines of influenza, respectively.

There were four studies reported the details of vaccine adjuvants. Among them three used AS03 adjuvant, and one had MF59 adjuvant. The pooled background rate of GBS was 5.40 per million (95%CI: 3.54 to 7.26 per million) for vaccine with AS03 adjuvant.

### Publication Bias and Sensitivity Analyses

**Figure 3:**
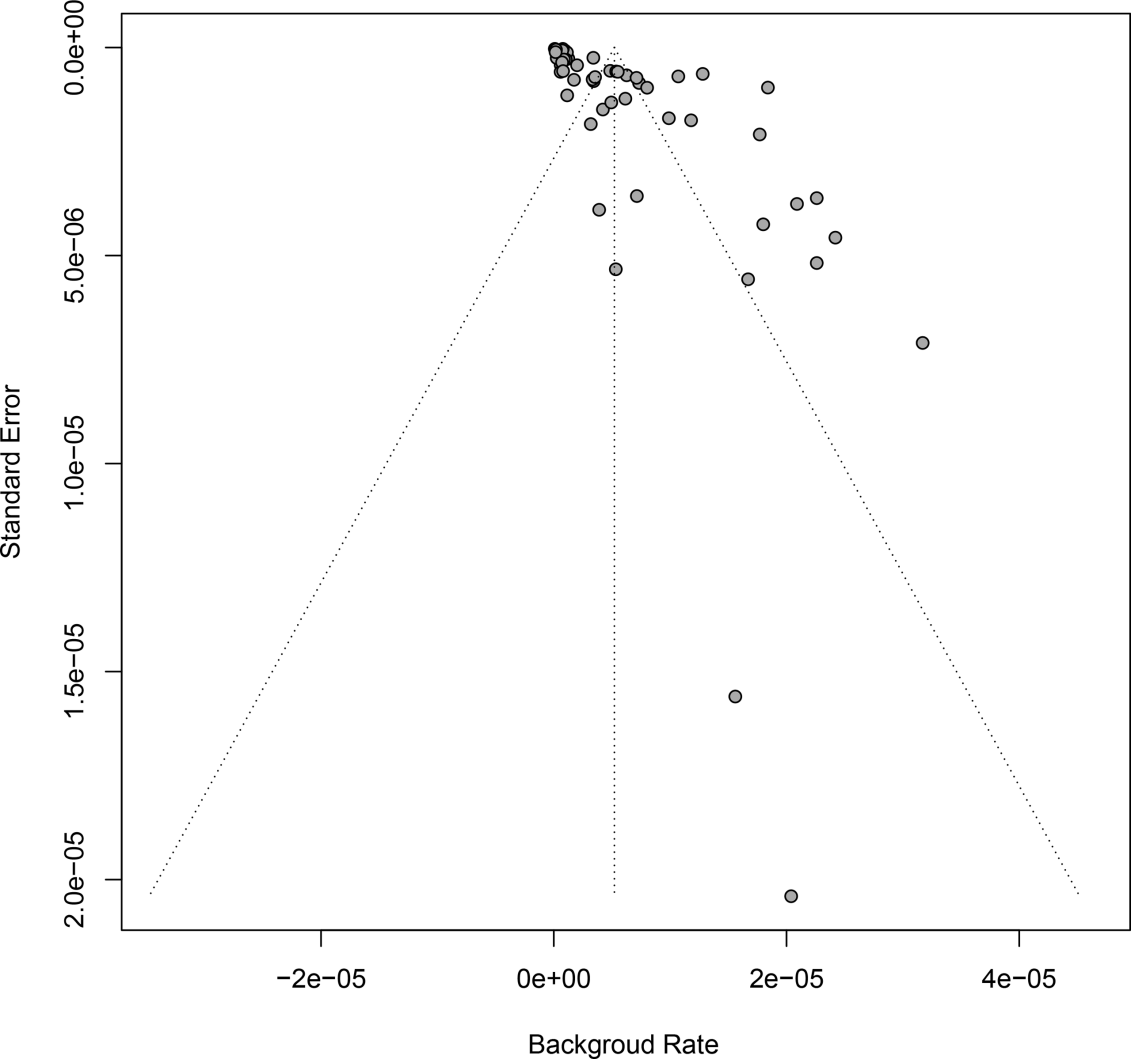
Funnel plot for publication bias analysis.

Funnel plot analysis and Egger’s test were used to examine the significance of publication bias underlying our study, by which a statistical significance was identified (**Table 2** and **Figure 3**). In order to control publication bias the trim-fill method was further performed, by which the pooled GBS rate was 1.71 per million (95% CI: 0 to 3.96 per million) after immunization of vaccine against virus, and 1.89 per million (95% CI: 1.48 to 2.30 per million) in 6 weeks follow-up after immunization.

To examine the strength of the pooled results, we performed a sensitivity analysis by omitting one study at a time. Consequently, the pooled result was not dominantly affected by any of the individual studies (**Figure S1**), indicating high stability of our results.

## Discussion

To the best of our knowledge, this study is the first to comprehensively summarize the incidence rates of GBS following mass immunizations of viral vaccines. Our meta-analyses, involving 58 original studies and 2,110,441,600 participants, identified a pooled rate 5.29 per million (95% CI:3.66 to 6.93 per million) among people received viral vaccines, and a pooled rate 3.09 per million (95% CI:2.67 to 3.51 per million) in 6 weeks of vaccination, equally 2.47 per 100,000 person-year (95%CI: 2.14 to 2.81 per 100,000 person-year). There was no significant increase in GBS incidence among population received viral vaccines compared to general population without prior vaccination. Subgroup analyses released the pooled rates of 2.77 per million (95%CI: 2.47 to 3.07 per million) for individuals received influenza vaccine and 2.44 per million (95%CI: 0.97 to 3.91 per million) for HPV vaccinees, respectively.

GBS is a demyelinating transient neurological disorder characterized by lack of paralysis and sensory impairment. GBS is an immune-related disorder, in which the immune response generates antibodies that cross-react with gangliosides (*i.e.*, GM1, GD1a, GT1b and GQ1b) at nerve membranes (75). This autoimmune response results in nerve damage or functional blockade of nerve conduction (76). Aberrant active immunization induced by artificial vaccines, hypothetically, is able to stimulate the immune system to produce specific antibodies, which contribute to cross reaction with epitopes on myelin or axons, leading to nerve damage (77). Vaccines might, as understood, damage the peripheral nerves directly (78). However, the causal associations between vaccines and GBS have not been substantially proved, *i.e.*, the association might not be causally established.

The mass immunization against COVID-19 has started unprecedentedly on a global scale. Recently, coincident GBS case was observed after administrated with COVID-19 vaccine (19). New considerations about vaccine safety will undoubtedly arise. Toward the public, it is critical to distinguish events that are temporally associated with vaccination from those directly caused by vaccines. Misinterpretation of GBS incidence that is only temporally coincident with but not caused by vaccination will not only obstruct the success of mass vaccination, but also hinder the development of newer vaccines (9).

During the 1976-77 A/H1N1 influenza immunization campaign, an increase of GBS was reported after vaccine administration (15), which suspended the immunization program temporarily, and initiated vaccine safety concerns. In the 1993-1994 influenza seasons, public concern of vaccine-related GBS arose again due to the increment of GBS (79). The 2009 H1N1 influenza pandemic motivated H1N1 vaccine campaigns in North America and Europe, where post-vaccination GBS concern was raised consequently (29). However, in 2009-2010, a surveillance of H1N1 influenza vaccine in 45 million persons showed a lower excess risk for GBS during the immunization campaign compared to earlier vaccination (73). In France, a study did not support the causation between GBS and H1N1 vaccination (80). Our pooled results show that the temporal coincidence of GBS in influenza vaccinees is not higher than that among general populations unvaccinated.

With regard to HPV, the debate on vaccine safety still exists, which remains one of the barriers to achievement of intensive global vaccination coverage. The Vaccine Adverse Event Reporting System (VAERS) in the United States reported a GBS rate of 0.2 per 100,000 dosages coincided with HPV vaccination from 2006 to 2008 (24). In a school-based HPV study in Canada, the overall background rate was 0.73/100,000 person-year for adolescents aged 7-17 years (81). Similarly, our study did not observe an increase in background rate of GBS after HPV vaccine administration.

The adjuvants of vaccines could affect the magnitude and quality of immune response. The AS03 adjuvant contains α-tocopherol, which might promote immune system activation in the nonregional lymph nodes (82), whereas MF59 might modulate cellular immune response at the injection site or regional lymph nodes (83). Moreover, an *in vitro* study demonstrated that α-tocopherol can raise the expression of hypocretin, leading to antigen presentation *via* human leukocyte antigens (84), which results in an autoimmune response, and damages hypocretin-producing neurons. In this current study, both vaccines adjuvanted with AS03 and those with MF59 have lower background rates of GBS than that of general populations, even though the background rate among individuals received vaccines with AS03 adjuvant is higher than that with MF59 adjuvant.

Our study has potential limitations that usually exist in observational studies and systematic reviews. Firstly, we aimed to summary data from studies that reported background rate of GBS in mass immunizations, which reflected the temporally coincidence of GBS in “real world”. In accordance with the predefined protocol, we did not include clinical trials that evaluate the safety of vaccines. Secondly, there was heterogeneity across original studies, which might limit the consolidation of the findings. Thirdly, most of original studies involved Caucasian participants, limiting the representability of our findings for other ethnic groups. Fourthly, as all the original studies are based on vaccine surveillance data, the methodological quality was not able to be evaluated. Fifthly, the increase in GBS reported in vaccination campaigns might result from the higher detection rate among vaccinees and increased reporting levels of GBS cases following receipt of vaccines.

In conclusion, our findings evidenced a mild increase in coincidental GBS during virus vaccination. We presented a reference for evaluation of the coincidental occurrence of GBS in mass vaccination campaigns, including SARS-CoV-2 vaccine.

## Data Availability Statement

The original contributions presented in the study are included in the article/**Supplementary Material**. Further inquiries can be directed to the corresponding authors.

## Author Contributions

HH, YXW, and WW designed this study. FW, DW, YJW, and CL contributed to literature search, review, data extraction. YLZ, ZG, PL, and YCZ conducted statistical analyses. FW, DW, YJW, and CL wrote the manuscript. HH, YXW, and WW contributed to manuscript revision. All authors have reviewed and approved the final version of this manuscript.

## Conflict of Interest

The authors declare that the research was conducted in the absence of any commercial or financial relationships that could be construed as a potential conflict of interest.

## Publisher’s Note

All claims expressed in this article are solely those of the authors and do not necessarily represent those of their affiliated organizations, or those of the publisher, the editors and the reviewers. Any product that may be evaluated in this article, or claim that may be made by its manufacturer, is not guaranteed or endorsed by the publisher.

## Supplementary Material

Supplementary Figure 1 Forest plot of **s**ensitivity analysis.CI, confidence interval.
