# Supplementary figures and images for: Population-Based Incidence of Guillain-Barré Syndrome During Mass Immunization With Viral Vaccines: A Pooled Analysis
Source: Front Immunol. 2022 Feb 3;13:782198. doi: 10.3389/fimmu.2022.782198 (PMC8850251; doi:10.3389/fimmu.2022.782198)

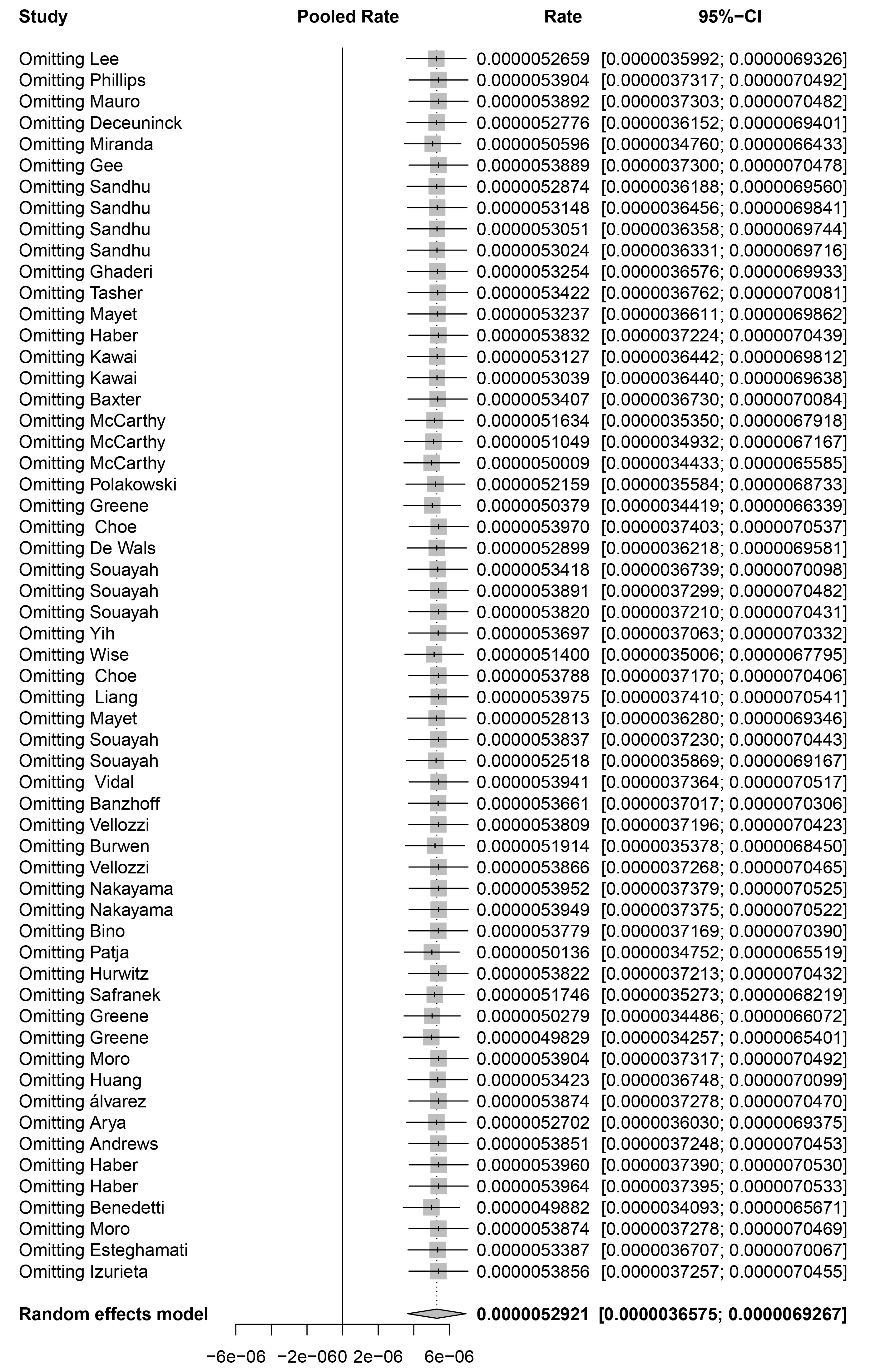

Supplement: Supplementary Figure 1 — Forest plot of sensitivity analysis.CI, confidence interval. [file Image_1.tif]
