# Supplementary material for: Population-Based Incidence of Guillain-Barré Syndrome During Mass Immunization With Viral Vaccines: A Pooled Analysis
Source: Front Immunol. 2022 Feb 3;13:782198. doi: 10.3389/fimmu.2022.782198 (PMC8850251; doi:10.3389/fimmu.2022.782198)
Supplement: Supplementary file 2 [file DataSheet_1.docx]

**[Supplementary materials]**

**Table S1 Checklist of PRISMA Statement**

| **Section/Topic** | **Checklist Item** | **Reported or not** |
| --- | --- | --- |
| TITLE |  |  |
| Title | Identify the report as a systematic review, meta-analysis, or both. | Y |
| ABSTRACT |  |  |
| Structured summary | Provide a structured summary including, as applicable: background; objectives; data sources; study eligibility criteria, participants, and interventions; study appraisal and synthesis methods; results; limitations; conclusions and implications of key findings; systematic review registration number. | Y |
| INTRODUCTION |  |  |
| Rationale | Describe the rationale for the review in the context of what is already known. | Y |
| Objectives | Provide an explicit statement of questions being addressed with reference to participants, interventions, comparisons, outcomes, and study design (PICOS). | Y |
| METHODS |  |  |
| Protocol and registration | Indicate if a review protocol exists, if and where it can be accessed (e.g., Web address), and, if available, provide registration information including registration number. | N |
| Eligibility criteria | Specify study characteristics (e.g., PICOS, length of follow-up) and report characteristics (e.g., years considered, language, publication status) used as criteria for eligibility, giving rationale. | Y |
| Information sources | Describe all information sources (e.g., databases with dates of coverage, contact with study authors to identify additional studies) in the search and date last searched. | Y |
| Search | Present full electronic search strategy for at least one database, including any limits used, such that it could be repeated. | Y |
| Study selection | State the process for selecting studies (i.e., screening, eligibility, included in systematic review, and, if applicable, included in the meta-analysis). | Y |
| Data collection process | Describe method of data extraction from reports (e.g., piloted forms, independently, in duplicate) and any processes for obtaining and confirming data from investigators. | Y |
| Data items | List and define all variables for which data were sought (e.g., PICOS, funding sources) and any assumptions and simplifications made. | Y |
| Risk of bias in individual studies | Describe methods used for assessing risk of bias of individual studies (including specification of whether this was done at the study or outcome level), and how this information is to be used in any data synthesis. | Y |
| Summary measures | State the principal summary measures (e.g., risk ratio, difference in means). | Y |
| Synthesis of results | Describe the methods of handling data and combining results of studies, if done, including measures of consistency (e.g., I2) for each meta-analysis. | Y |
| Risk of bias across studies | Specify any assessment of risk of bias that may affect the cumulative evidence (e.g., publication bias, selective reporting within studies). | Y |
| Additional analyses | Describe methods of additional analyses (e.g., sensitivity or subgroup analyses, meta-regression), if done, indicating which were pre-specified. | Y |
| RESULTS |  |  |
| Study selection | Give numbers of studies screened, assessed for eligibility, and included in the review, with reasons for exclusions at each stage, ideally with a flow diagram. | Y |
| Study characteristics | For each study, present characteristics for which data were extracted (e.g., study size, PICOS, follow-up period) and provide the citations. | Y |
| Risk of bias within studies | Present data on risk of bias of each study and, if available, any outcome-level assessment (see Item 12). | Y |
| Results of individual studies | For all outcomes considered (benefits or harms), present, for each study: (a) simple summary data for each intervention group and (b) effect estimates and confidence intervals, ideally with a forest plot. | Y |
| Synthesis of results | Present results of each meta-analysis done, including confidence intervals and measures of consistency. | Y |
| Risk of bias across studies | Present results of any assessment of risk of bias across studies (see Item 15). | Y |
| Additional analysis | Give results of additional analyses, if done (e.g., sensitivity or subgroup analyses, meta-regression [see Item 16]). | Y |
| DISCUSSION |  | Y |
| Summary of evidence | Summarize the main findings including the strength of evidence for each main outcome; consider their relevance to key groups (e.g., health care providers, users, and policy makers). | Y |
| Limitations | Discuss limitations at study and outcome level (e.g., risk of bias), and at review level (e.g., incomplete retrieval of identified research, reporting bias). | Y |
| Conclusions | Provide a general interpretation of the results in the context of other evidence, and implications for future research. | Y |
| FUNDING |  |  |
| Funding | Describe sources of funding for the systematic review and other support (e.g., supply of data) | Y |

Y: the item was reported in article, N: the item was not reported.

**Table S****2 Scale for quality assessment based on PRISMA statement and MOOSE guideline**

| **Criteria** | **Score** |
| --- | --- |
| **Representativeness of participants** |  |
| Characteristics of participants were described. | 1 |
| Consecutive/randomly selected from case population was clearly defined. | 1 |
| Can we be confident in the assessment of exposure? | 1 |
| The percentage of loss to follow-up was provided, or the reasons of loss to follow-up were mentioned. | 1 |
| **Accuracy of information** |  |
| Methods of variable measurement were offered | 1 |
| Definitions of outcome were offered. | 1 |
| **Statistical analysis** |  |
| Methods of statistical analyses were adequate to resolve research hypothesis. | 1 |
| Multivariate analyses were performed if necessary. | 1 |
| **Final question** |  |
| If there were any other important flaws in the design, the study would be not included. |  |

**Table S3 Incidence of Guillain-Barré syndrome (GBS) in general population**

| **Study (author)** | **County** | **Number of GBS cases** | **Incidence rate (per 100,000 person-year)** |
| --- | --- | --- | --- |
| Chio et al.(1) | Italy | 126 | 1.44 |
| Govoni et al.(2) | Italy | 69 | 1.89 |
| Bogliun and Beghi(3) | Italy | 138 | 1.55 |
| Cuadrado et al.(4) | Spain | 98 | 1.25 |
| Deceuninck et al.(5) | Canada | 33 | 0.81 |
| Chen et al.(6) | China | 534 | 0.59 |
| Rivera-Lillo et al.(7) | Chile | 4,158 | 2.1 |
| Delannoy et al.(8) | France | 9391 | 2.42 |
| Sipilä et al.(9) | Finland | 917 | 1.70 |
| Matsui et al. (10) | Japan | 71 | 0.42 |
| Levison et al.(11) | Denmark | 2,319 | 1.77 |
| Stojanov et al. (12) | Serbia | 640 | 1.1 |
| Aragonès et al.(13) | Spain | 43 | 2.07 |
| Ah-Young Kim et al. (14) | South Korea | 941 | 1.82 |
| Chiesa et al.(15) | Uruguayan | 51 | 1.7 |
| Kasemsap et al.(16) | Thailand | 4521 | 0.48-0.93 |
| Tonekaboni et al. (17) | Iranian | 1884 | 1.72 |

**Reference**

1. Chiò A, Cocito D, Leone M, Giordana MT, Mora G, Mutani R. Guillain-Barré syndrome: a prospective, population-based incidence and outcome survey. *Neurology* (2003) 60(7):1146-50. Epub 2003/04/12. doi: 10.1212/01.wnl.0000055091.96905.d0. PubMed PMID: 12682322.

2. Govoni V, Granieri E, Manconi M, Capone J, Casetta I. Is there a decrease in Guillain-Barré syndrome incidence after bovine ganglioside withdrawal in Italy? A population-based study in the Local Health District of Ferrara, Italy. *J Neurol Sci* (2003) 216(1):99-103. Epub 2003/11/11. doi: 10.1016/s0022-510x(03)00215-6. PubMed PMID: 14607309.

3. Bogliun G, Beghi E. Incidence and clinical features of acute inflammatory polyradiculoneuropathy in Lombardy, Italy, 1996. *Acta Neurol Scand* (2004) 110(2):100-6. Epub 2004/07/10. doi: 10.1111/j.1600-0404.2004.00272.x. PubMed PMID: 15242417.

4. Cuadrado JI, de Pedro-Cuesta J, Ara JR, Cemillán CA, Díaz M, Duarte J, et al. Public health surveillance and incidence of adulthood Guillain-Barré syndrome in Spain, 1998-1999: the view from a sentinel network of neurologists. *Neurol Sci* (2004) 25(2):57-65. Epub 2004/06/29. doi: 10.1007/s10072-004-0231-6. PubMed PMID: 15221623.

5. Deceuninck G, Boucher RM, De Wals P, Ouakki M. Epidemiology of Guillain-Barré syndrome in the province of Quebec. *Can J Neurol Sci* (2008) 35(4):472-5. Epub 2008/11/01. doi: 10.1017/s0317167100009136. PubMed PMID: 18973064.

6. Chen Y, Ma F, Zhang J, Chu X, Xu Y. Population incidence of Guillain-Barré syndrome in parts of China: three large populations in Jiangsu province, 2008-2010. *European journal of neurology* (2014) 21(1):124-9. Epub 2013/10/10. doi: 10.1111/ene.12265. PubMed PMID: 24102733.

7. Rivera-Lillo G, Torres-Castro R, Burgos PI, Varas-Díaz G, Vera-Uribe R, Puppo H, et al. Incidence of Guillain-Barré syndrome in Chile: a population-based study. *Journal of the peripheral nervous system : JPNS* (2016) 21(4):339-44. Epub 2016/08/02. doi: 10.1111/jns.12182. PubMed PMID: 27477441.

8. Delannoy A, Rudant J, Chaignot C, Bolgert F, Mikaeloff Y, Weill A. Guillain-Barré syndrome in France: a nationwide epidemiological analysis based on hospital discharge data (2008-2013). *Journal of the peripheral nervous system : JPNS* (2017) 22(1):51-8. Epub 2016/12/20. doi: 10.1111/jns.12202. PubMed PMID: 27991707.

9. Sipilä JOT, Soilu-Hänninen M, Ruuskanen JO, Rautava P, Kytö V. Epidemiology of Guillain-Barré syndrome in Finland 2004-2014. *Journal of the peripheral nervous system : JPNS* (2017) 22(4):440-5. Epub 2017/11/03. doi: 10.1111/jns.12239. PubMed PMID: 29095548; PubMed Central PMCID: PMCPMC5765466.

10. Matsui N, Nodera H, Kuzume D, Iwasa N, Unai Y, Sakai W, et al. Guillain-Barré syndrome in a local area in Japan, 2006-2015: an epidemiological and clinical study of 108 patients. *European journal of neurology* (2018) 25(5):718-24. Epub 2018/01/18. doi: 10.1111/ene.13569. PubMed PMID: 29337417.

11. Levison LS, Thomsen RW, Christensen DH, Mellemkjær T, Sindrup SH, Andersen H. Guillain-Barré syndrome in Denmark: validation of diagnostic codes and a population-based nationwide study of the incidence in a 30-year period. *Clinical epidemiology* (2019) 11:275-83. Epub 2019/05/23. doi: 10.2147/clep.S199839. PubMed PMID: 31114387; PubMed Central PMCID: PMCPMC6497480.

12. Stojanov A, Berisavac I, Bozovic I, Arsenijevic M, Lukic-Rajic S, Petrovic M, et al. Incidence and mortality rates of Guillain-Barré syndrome in Serbia. *Journal of the peripheral nervous system : JPNS* (2020) 25(4):350-5. Epub 2020/09/02. doi: 10.1111/jns.12412. PubMed PMID: 32869405.

13. Aragonès JM, Altimiras J, Alonso F, Celedón G, Alfonso S, Roura P, et al. Incidence and clinical characteristics of Guillain-Barré syndrome in Osona (Barcelona, Spain), 2003-2016. *Neurologia (Barcelona, Spain)* (2021) 36(7):525-30. Epub 2021/09/20. doi: 10.1016/j.nrleng.2018.03.020. PubMed PMID: 34537166.

14. Kim AY, Lee H, Lee YM, Kang HY. Epidemiological Features and Economic Burden of Guillain-Barré Syndrome in South Korea: A Nationwide Population-Based Study. *Journal of clinical neurology (Seoul, Korea)* (2021) 17(2):257-64. Epub 2021/04/10. doi: 10.3988/jcn.2021.17.2.257. PubMed PMID: 33835747; PubMed Central PMCID: PMCPMC8053545.

15. Chiesa M, Decima R, Bertinat A, Poggi L, Hackembruch H, Montenegro C, et al. Incidence of Guillain-Barré syndrome in an Uruguayan population. A prospective cohort study. *Journal of the peripheral nervous system : JPNS* (2021) 26(2):209-15. Epub 2021/05/05. doi: 10.1111/jns.12450. PubMed PMID: 33945181.

16. Kasemsap N, Vorasoot N, Kongbunkiat K, Tiamkao S, Chotmongkol V, Sawanyawisuth K, et al. The epidemiology of Guillain-Barré syndrome in Thailand over 13 years (2005-2017): A nationwide population-based retrospective cohort study. *Journal of the peripheral nervous system : JPNS* (2021) 26(2):202-8. Epub 2021/05/11. doi: 10.1111/jns.12453. PubMed PMID: 33969589.

17. Tonekaboni SH, Mahmoudi S, Abdollah Gorji F, Nejad Biglari H, Taghdiri MM, Etemadi K, et al. Epidemiology of Guillain-Barré Syndrome in Iranian Children Aged 0-15 Years (2008-2013). *Iranian journal of child neurology* (2021) 15(4):27-34. Epub 2021/11/17. doi: 10.22037/ijcn.v15i4.25087. PubMed PMID: 34782839; PubMed Central PMCID: PMCPMC8570621.
